# Lipoprotein(a) as a Predictor of Cardiovascular Risk in Acute Coronary Syndrome Patients Undergoing Percutaneous Coronary Intervention: A Systematic Review

**DOI:** 10.31083/RCM42784

**Published:** 2025-09-22

**Authors:** Azad Mojahedi, On Chen, Hal A Skopicki, Tahmid Rahman, Amirhossein Sadeghian

**Affiliations:** ^1^Department of Internal Medicine, Stony Brook University Hospital, Stony Brook, NY 11794, USA; ^2^Division of Cardiology, Stony Brook University Hospital, Stony Brook, NY 11794, USA; ^3^School of Medicine, Zabol University of Medical Sciences, 98914 Zabol, Sistan and Baluchestan, Iran

**Keywords:** lipoprotein(a), acute coronary syndrome, percutaneous coronary intervention, cardiovascular events

## Abstract

**Background:**

Despite advancements in treatment, coronary artery disease (CAD) remains a significant global health concern. Although lipoprotein(a) [Lp(a)] is recognized as a crucial cardiovascular risk factor associated with increased risk, the prognostic value of using Lp(a) levels in patients with acute coronary syndrome (ACS) who have undergone percutaneous coronary intervention (PCI) remains debatable. This review aimed to investigate the association between Lp(a) levels and recurrent ischemic events in patients with ACS undergoing PCI.

**Methods:**

This systematic review included studies with individuals aged ≥18 years diagnosed with ACS who underwent PCI and had Lp(a) measurements. The included studies were sourced from the PubMed database, with a focus on articles published between January 2020 and January 2025. Keywords related to Lp(a) and cardiovascular diseases were used in the search. Data extraction involved a review of titles and abstracts followed by quality assessment using the QUADAS-2 tool.

**Results:**

The final analysis included 10 studies with a combined population of 20,896 patients from diverse regions, including Japan, India, Egypt, China, and South Korea. Key findings indicate that elevated Lp(a) levels are significantly associated with adverse cardiovascular outcomes, including myocardial infarction and mortality, both in hospital and during long-term follow-up.

**Conclusions:**

This review highlights Lp(a) as a critical biomarker for predicting recurrent cardiovascular events in ACS patients post-PCI. The consistent correlation between elevated Lp(a) levels and adverse outcomes underscores the necessity of routine monitoring and targeted management of Lp(a) to mitigate residual cardiovascular risk.

## 1. Introduction

Despite significant progress in invasive and medical treatments over the past three decades, coronary artery disease (CAD) remains a major contributor to global morbidity and mortality [1]. A significant risk factor for CAD is dyslipidemia. Research has demonstrated that reducing cardiovascular events after percutaneous coronary intervention (PCI) can be achieved by decreasing serum low-density lipoprotein cholesterol (LDL-C) levels through the use of statin medications [2]. Despite intensive statin therapy, a considerable proportion of patients continue to experience adverse cardiovascular outcomes including myocardial infarction (MI), in-stent thrombosis, and cardiovascular-related mortality. The issues of continuing risk have also been well established by a variety of randomized clinical trials; many existing patient populations treated with aggressive lipid-lowering strategies continue to experience the burden of cardiovascular complications [3, 4].

Traditional lipid-altering therapy, targeting LDL-C levels, including statin medications, has been shown to be inadequate, and conflicting studies have demonstrated that elevations in lipoproteins result in cardiovascular events [5, 6]. Lipoprotein(a) [Lp(a)] is an LDL-like particle generated in the liver, and its serum concentration is primarily driven by the number of kringle IV type 2 protein domain repeats. Most studies have demonstrated that elevated serum Lp(a) levels are associated with increased cardiovascular risk [7, 8]. Unfortunately, statin therapy is not effective in lowering Lp(a) levels [9]. There is substantial variation in the expression of Lp(a) across racial and ethnic populations. Most observational and epidemiological studies have focused primarily on Caucasians [10, 11]. Evidence is becoming increasingly consistent that proprotein convertase subtilisin/kexin type 9 (PCSK9) inhibitors lower Lp(a) levels in a manner similar to that of cardiovascular risk. Currently, Lp(a) is considered a modifiable risk factor for cardiovascular conditions. This has led to some speculation that Lp(a) reduction could reduce remaining cardiovascular risk [1, 7, 12].

Globally, Lp(a) has been established as an indicator of cardiovascular disease. The continually-developing guidelines recommend checking Lp(a) only once to identify individuals at significant risk of cardiovascular disease. The prognostic significance of Lp(a) levels in patients with a history of cardiovascular events, especially in patients with acute coronary syndrome (ACS), remains unclear. Evidence continues to demonstrate conflicting results regarding the risk of Lp(a). Where Lp(a) has been predictive, there is evidence that Lp(a) does not retain the same predictive value for different facets of recurrent cardiovascular events. This study examined Lp(a) levels with respect to recurrent ischemic events in patients with ACS after PCI.

## 2. Material and Methods

### 2.1 Search Strategy

A comprehensive analysis was performed to examine the influence of Lp(a) levels on residual cardiovascular events in ACS patients undergoing PCI. The investigation utilized the PubMed database, focusing on the period from January 2020 to January 2025. We limited the search period to the last five years to focus on the most recently published evidence and contemporary PCI practice, though this restriction may have omitted relevant earlier work. The Advanced Search Builder was employed, with keyword searches limited to [Title OR Abstract]. The study included only English-language research articles to ensure consistency in data extraction and interpretation, though this represents a potential limitation regarding language bias. We used a combination of keywords and medical subject headings (MeSH) tailored to each database. The search terms incorporated were: ‘(Lipoprotein(a)) AND (Acute Coronary Syndrome) AND (Percutaneous Coronary Intervention) AND (Cardiovascular Disease)’.

### 2.2 Inclusion and Exclusion Criteria

This systematic review considered original studies involving individuals aged 18 years or older diagnosed with ACS who underwent PCI and had Lp(a) measurements. Additional relevant literature was identified through reference checks of the selected studies. Exclusion criteria included studies with participants who had coronary artery bypass grafting, were critically ill requiring mechanical circulatory support or ventilation, did not successfully receive coronary artery stenting, or died before PCI. Furthermore, this review excluded case reports and series with few patients, review articles without original data, editorials, letters, and conference papers. References within chosen research were examined for other pertinent literature.

### 2.3 Data Extraction 

The evaluation of titles and abstracts was performed by two authors (AM and OC). Subsequently, data were extracted from studies that met the inclusion and exclusion criteria in accordance with the survey specifications.

We incorporated relevant studies identified through a review of the reference lists of previously published review articles. Ten eligible research articles in their final form were selected for inclusion. In some cases, we opted to focus solely on the primary findings aligned with the objectives of this review.

### 2.4 Quality Assessment

Two authors (AM and TR) independently assessed the quality of the published interventions. A third author (HAS) ensured that any disagreements were resolved. To determine the possibility of bias in each of the included studies, the QUADAS-2 instrument was utilized to evaluate the population, technique, analysis, and reporting requirements of each study [13]. The tool comprises four main categories: flow and timing, reference standards, index tests, and patient selection. For each specific study, every category was evaluated as either “low”, “high”, or “unclear”. Then, the ratings for every domain were shown, along with a subjective judgment of the overall quality of the included studies.

## 3. Results

### 3.1 Study Selection and Studies Characteristics 

**Fig. 1.**
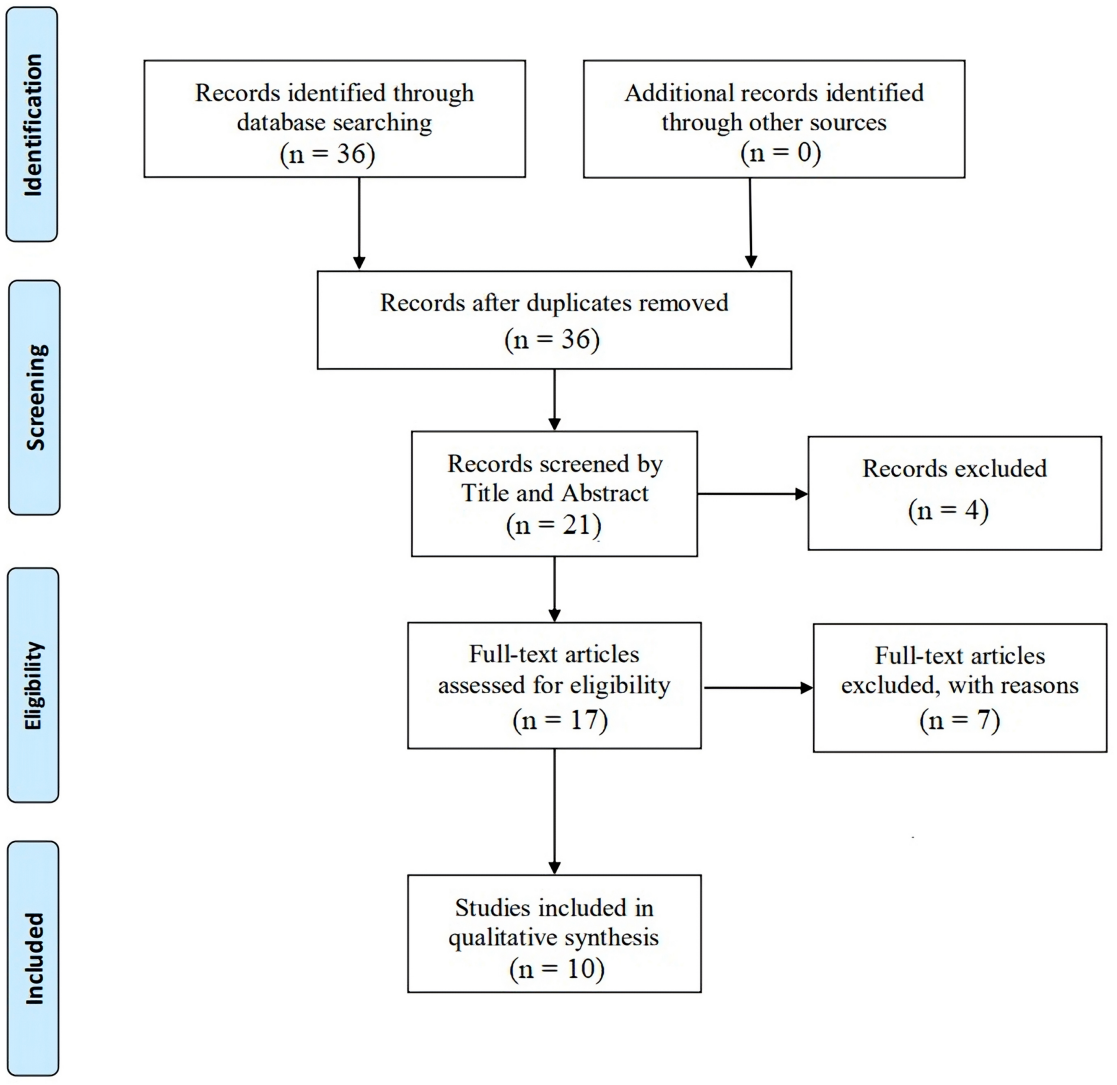
**Preferred Reporting Items for Systematic reviews and Meta-Analyses (PRISMA) flow diagram for enrollment of studies**.

After conducting a thorough search, we found 36 articles by January 2025. Following title and abstract screening, 15 articles were excluded, and 21 articles were retained for further analysis. After screening, we excluded 4 studies and were left with 17 to assess their full texts. This systematic review included ten studies. The process for selecting these studies is illustrated in Fig. 1. The total study population across these studies is 20,896 patients, with research conducted in various regions including Japan, India, Egypt, China, and South Korea. The studies highlight the significant implications of elevated Lp(a) levels on adverse cardiac outcomes, suggesting that monitoring and potentially lowering Lp(a) could be important in managing cardiovascular risk in these patients. We extracted data from ten eligible articles, all prospective studies, and summarized the information in Table 1 (Ref. [14, 15, 16, 17, 18, 19, 20, 21, 22]).

**Table 1.** **Summary of studies on lipoprotein(a) levels and cardiovascular events in patients undergoing percutaneous coronary intervention**.

Year	Study type	Study population	Mean of age, year ± SD	Gender, male (%)	Mean of Lp(a)	Cut-off value of Lp(a) (defined as high Lp(a))	Mean follow-up	Conclusion	Reference
2024	Retrospective study	249	66.6 ± 12.6	79.0%	19.0 ± 21.8 mg/dL (pre-PCI)	N/A	≥1000 days	Lp(a) levels in ACS patients significantly declined post-emergent PCI, with a greater decrease from 0 to 12 hours [Lp(a)Δ0-12] independently linked to a poorer prognosis, including increased MACE incidence.	[14]
2024	Retrospective study	600	53.02 ± 9.7	79.16%	∼>30 mg/dL	≥30 mg/dL	18 months	Elevated Lp(a) levels are an independent risk factor for adverse cardiac events in patients with CAD undergoing PCI	[15]
2023	Prospective study	70	52.04 ± 9.4	90%	N/A	24.55 mg/dL	Hospitalization period	High plasma Lp(a) levels in STEMI patients can be used to predict severe adverse cardiac events, including acute heart failure, reinfarction, and in-hospital mortality.	[16]
2022	Retrospective study	488	65.9 ± 9.7	67.2%	Baseline: 13.0 mg/dL	≥50 mg/dL	31.4 months	Severe increases in Lp(a) following statin therapy raise the risk of MACE, whereas mild-to-moderate increases may not affect cardiovascular prognosis.	[17]
2021	Retrospective study	6309	60.1 ± 10.06	75.2%	13.0 mg/dL	>23 mg/dL	18 months	In ACS patients undergoing PCI, there was a synergistic effect between the GRACE risk score and on-statins Lp(a) levels on predicting cardiovascular events.	[18]
2021	Prospective study	12,064	∼61.84	72.6%	18.6 mg/dL	>30 mg/dL	7.4 years	Elevated levels of Lp(a) were significantly associated with recurrent ischemic events in patients who underwent PCI.	[19]
2020	Prospective study	4078	56.8 ± 10.5	76.5%	15.3 mg/dL	≥30 mg/dL	4.9 years	High Lp(a) levels (≥30 mg/dL) were associated with a significantly increased risk of cardiovascular events.	[20]
2021	Retrospective study	1292	56.5	69.1%	Low Lp(a) group: 17.6 mg/dL	30 mg/dL	N/A	High Lp(a) is associated with more severe coronary artery lesions and higher odds of congestive heart failure and composite in-hospital outcomes.	[21]
					High Lp(a) group: 73.6 mg/dL				
2020	Retrospective study	350	63.49 ± 10.85	80.3%	118.0 mmol/L	118.0 mmol/L	12 months	Elevated Lp(a) levels are associated with an increased incidence of revascularization in CAD patients after PCI with LDL-C goal attainment.	[22]

Lp(a), lipoprotein(a); ACS, acute coronary syndrome; PCI, percutaneous coronary intervention; MACE, major adverse cardiac events; STEMI, ST-elevation myocardial infarction; N/A, not applicable; CAD, coronary artery disease.

### 3.2 Quality Assessment

**Fig. 2.**
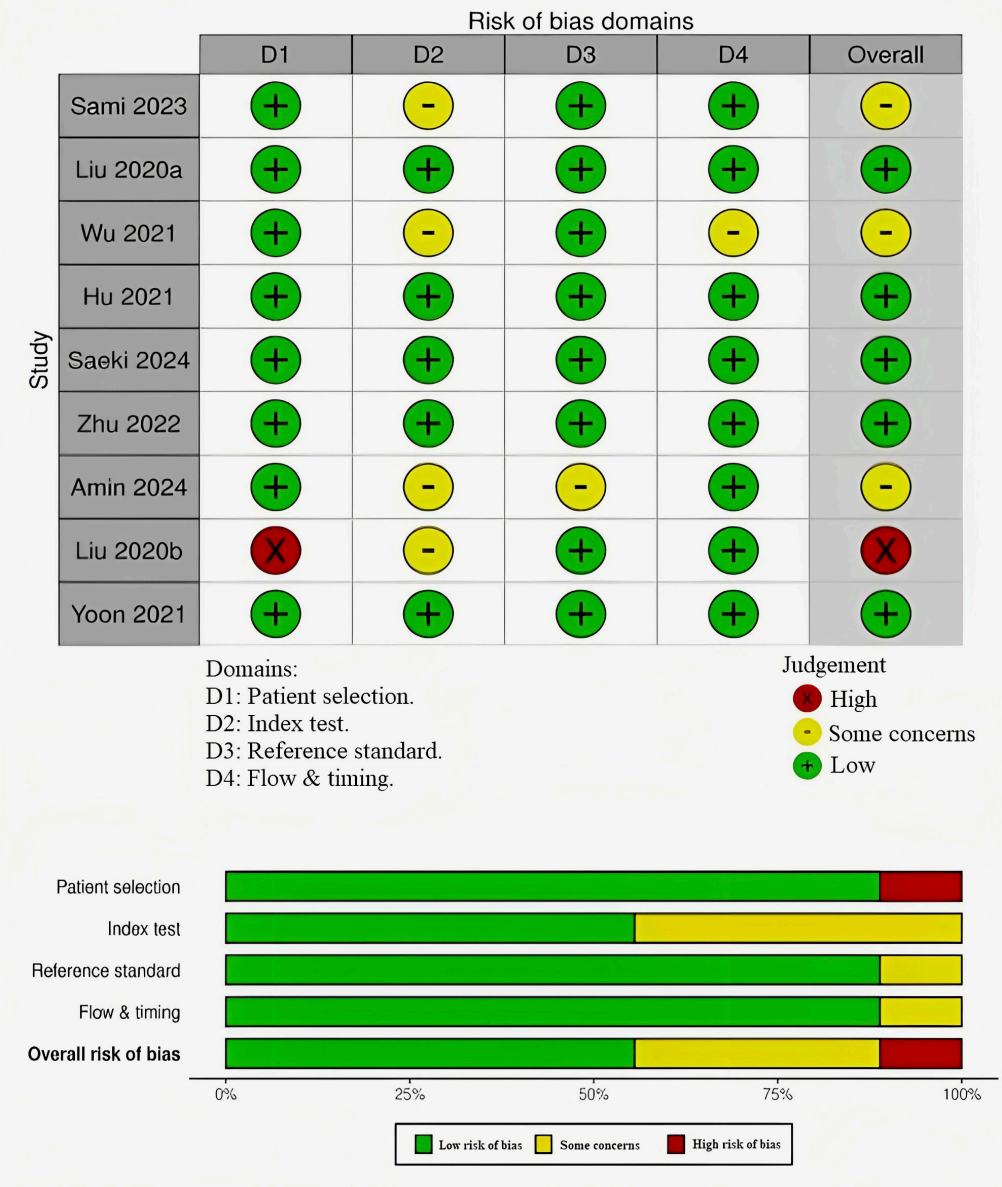
**Quality assessment and bias risk assessment in the investigations included in this review**.

Fig. 2 shows the risk of bias across various domains in the included studies. Only one study (Liu *et al*. [22]) was flagged with a high risk of bias, primarily due to issues with patient selection. This raises concerns regarding the representativeness of the study population. Several other studies (Sami *et al*. [16], Wu *et al*. [21], and Amin *et al*. [15]) have shown concerns in different domains. Sami *et al*. [16] and Wu *et al*. [21] had concerns regarding the index test, while Amin *et al*. [15] had concerns regarding both the index test and reference standard. The remaining studies Liu *et al*. [20], Hu *et al*. [18], Saeki *et al*. [14], Zhu *et al*. [17], and Yoon *et al*. [19]) were generally judged to have a low risk of bias across all domains.

## 4. Discussion

### 4.1 Structure and Metabolism of Lp(a) 

Lp(a) is a distinct lipoprotein, characterized by an LDL-like particle that is covalently linked to apolipoprotein(a) [apo(a)] via a disulfide bond with apolipoprotein B100 (apoB100). Lp(a) has a spherical shape, is 10% larger in diameter, and has approximately twice the molecular weight of LDL. Its primary protein is apoB, and it also contains varying amounts of albumin, apoC, and apoAIII. Its high carbohydrate content, particularly sialic acid, increases hydrated density [23, 24].

In contrast to LDL, the metabolism of Lp(a) is poorly understood and is largely controlled by genetic factors at the apo(a) locus, which confer high heritability and regulate hepatic apo(a) production. Lp(a) levels are also strongly affected by the liver’s synthesis of apoB100. It remains unknown where Lp(a) assembles from LDL and apo(a) and how it interacts with receptors *in vivo* [25, 26]. Although the exact mechanisms of clearance are unknown, its lipid and protein components may be eliminated by the hepatic scavenger receptor class B type I [25].

Essentially, the distinct structure, genetically determined plasma concentrations, and elusive assembly/clearance pathways of Lp(a) underscore the necessity of additional investigations to create treatments that reduce its cardiovascular hazards.

### 4.2 Pathophysiology of Lp(a)

The physiological role of Lp(a) is unknown [27], yet the structural resemblance between Lp(a) and plasminogen (PLG) insinuates that it may represent the mechanistic bridge linking cholesterol transport and the fibrinolytic system to favor wound healing and hemostasis [28]. Lp(a) probably transports cholesterol to the sites of injury for cell membrane repair and tissue regeneration [29, 30] and inhibits fibrinolysis to provide blood clot stability, thus preventing excessive bleeding [31, 32]. Nevertheless, these functions remain under scrutiny, as their mechanisms are not well understood [33].

**Fig. 3.**
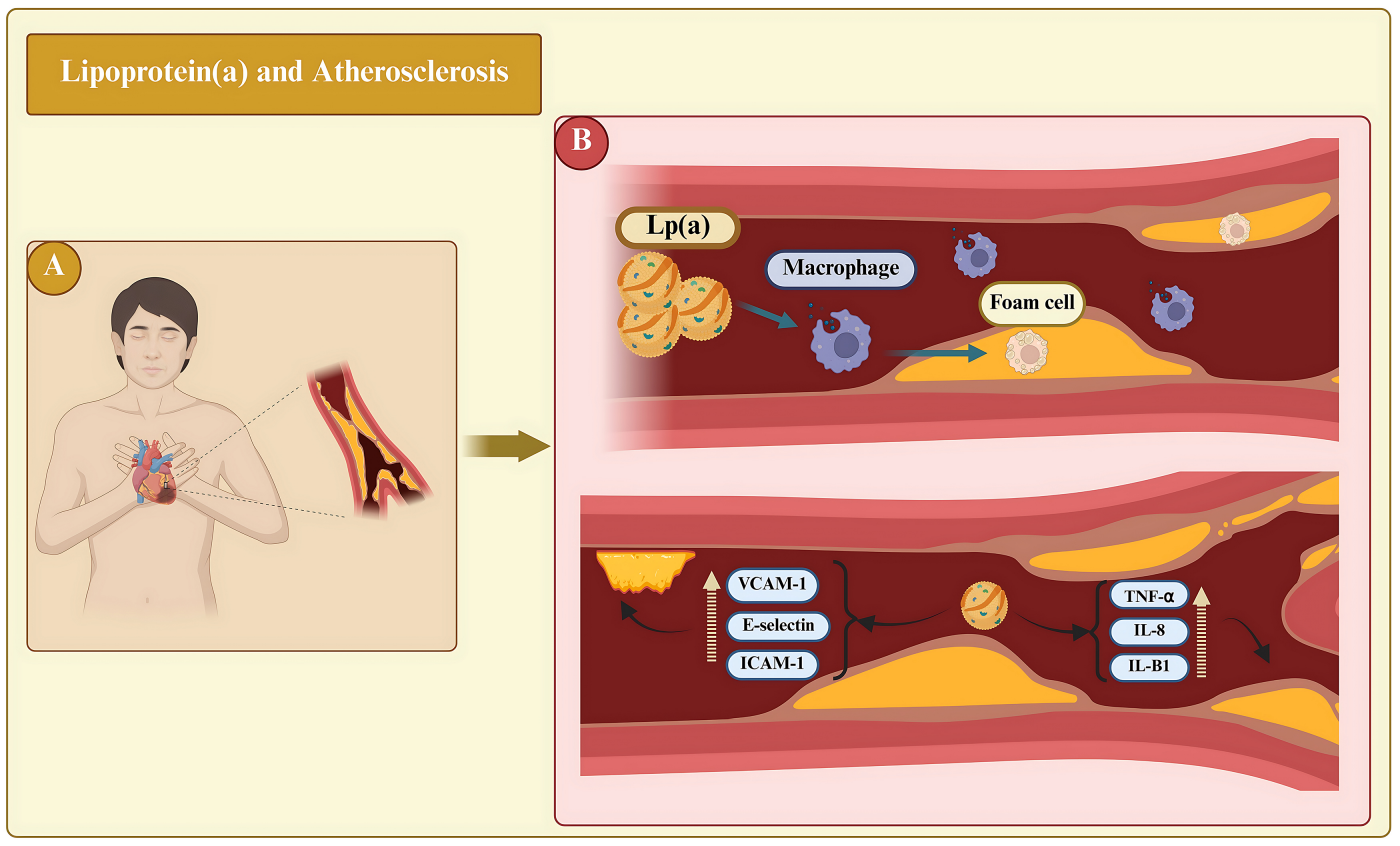
**The role of lipoprotein(a) [Lp(a)] on atherosclerotic processes and atherothrombosis is significant**. (A) Represents the systemic clinical perspective, showing a patient with cardiovascular disease in whom elevated Lp(a) levels circulate throughout the body and contribute to atherosclerotic disease. (B) Lp(a) penetrates vessel walls, promotes foam cell formation, and triggers inflammatory cascades. It depicts the upregulation (broken yellow upward arrow) of adhesion molecules and pro-inflammatory cytokines that enhance endothelial activation, monocyte transmigration, and plaque vulnerability, explaining the role of Lp(a) in atherothrombosis. TNF-α, tumor necrosis factor-α; IL, interleukin; ICAM1, intercellular adhesion molecule-1; VCAM-1, vascular cell adhesion molecule-1. Created with BioRender.com.

Elevated levels of Lp(a) constitute a major independent risk factor for cardiovascular disease (CVD), favoring atherogenesis, thrombosis, and inflammation (Fig. 3) [34]. These Lp(a) species have been shown *in vitro*and in animal models to convene the atherosclerotic process via pathways that include the stimulation of smooth muscle cell proliferation, formation of foam cells, and production of interleukin (IL)-8 [35]. It interacts with extracellular matrix components, including fibrin, fibronectin, and proteoglycans, to present cholesterol at sites of vascular injury for reparative processes [36, 37].

Oxidized phospholipids (OxPls) in plasma are covalently bound to Lp(a), and both are biomarkers of CVD [38, 39]. Lp(a) must enter and accumulate inside the artery and aortic valve leaflet intima to cause CVD [40]. This is entered into the vessel walls at a comparatively slower rate than LDL but is accelerated by injury sites by two to three times in a rabbit model [41, 42]. Lp(a) strongly binds to exposed fibrin and glycosaminoglycans and hence, may accumulate in contrast to other lipoproteins that contain apoB, fostering the pathogenesis of progressive aortic stenosis and complications after bypass [43].

### 4.3 Factors Affecting the Level of Lp(a) in Different Populations

Data from the UK Biobank showed that race and ethnicity significantly influenced Lp(a) concentrations. The study revealed distinct median values across different ethnic groups: white individuals, 19 nmol/L; South Asians, 31 nmol/L; black individuals, 75 nmol/L; and Chinese individuals, 16 nmol/L [44, 45]. Consequently, Lp(a) could significantly explain the differences in CVD rates among various ethnic groups. Studies encompassing diverse ethnic groups have demonstrated that variations in the *LPA* gene accounted for 17–77% of the variability in Lp(a) concentrations. Of this variation, 80% was linked to the number of kringle domains within Lp(a). These results indicate that genetic components are the main factors influencing Lp(a) levels, with minimal effects of age and sex [44, 46].

Several additional factors have been found to influence Lp(a) levels. Research has shown that Lp(a) concentrations can fluctuate in response to various forms of physical stress, such as sepsis, severe burns, acute coronary syndrome, and rheumatological conditions [47]. However, the nature of these changes remains controversial. Research indicates that Lp(a) levels decrease markedly during stress, whereas others report no change or even an elevation in Lp(a) levels under stressful conditions [39].

Research has indicated that Lp(a) levels increase in women following menopause. A comprehensive analysis of 15 investigations revealed that premenopausal women had lower Lp(a) concentrations compared to their postmenopausal counterparts, with an average difference of 3.77 mg/dL between the two groups. Interestingly, three studies examining Lp(a) plasma levels before and after bilateral oophorectomy found no significant change [48]. Additionally, studies suggest that hormone replacement therapy may reduce plasma Lp(a) levels [49]. The relationship between sex hormones and Lp(a) is intricate. Nonetheless, recent data show that Lp(a) levels increase after menopause and may be partially responsible for the increase in cardiovascular disease seen in women post-menopause [50].

The potential effect of aging on Lp(a) levels in blood plasma is not well understood. Limited studies suggest that aging may be associated with increased Lp(a). Studies assessing Lp(a) levels in different age groups have shown that Lp(a) levels are higher in older individuals [7, 51]. Furthermore, studies concerning Lp(a) levels in children have shown that Lp(a) levels can vary considerably, and a single value may lead to considerable underestimation of risk. Therefore, researchers suggest the repeated measurement of Lp(a) in adulthood rather than a single value for more appropriate treatment management [10, 52].

**Fig. 4.**
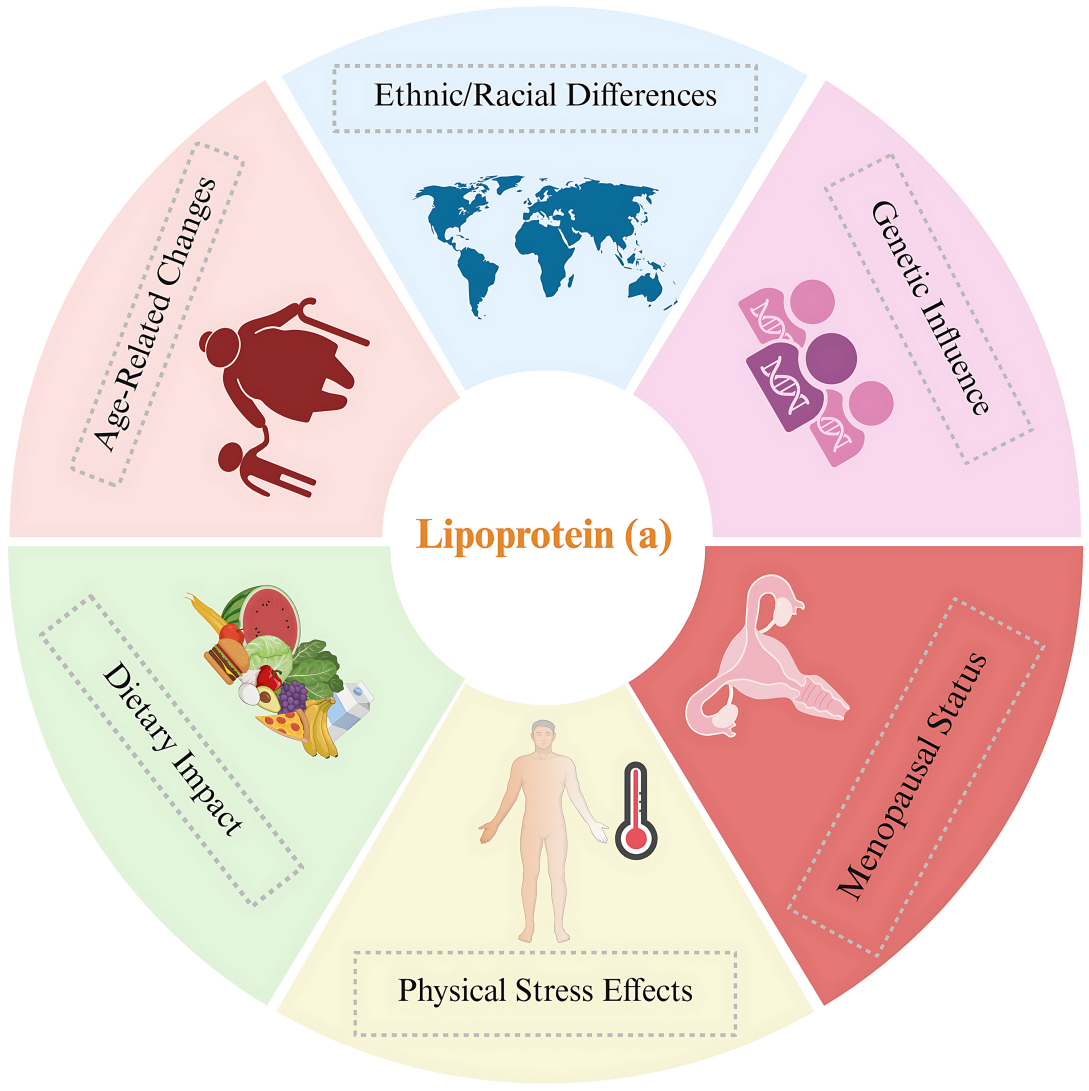
**Factors affecting Lipoprotein(a) levels in various populations**. Created with BioRender.com.

Research on the effects of diet and certain nutrients on Lp(a) has produced conflicting results. In two studies, Lp(a) increased with carbohydrate substitution for saturated fats, whereas other studies showed little to no effect. Similarly, replacing fats with monounsaturated or polyunsaturated fats has produced inconsistent results. Collectively, these findings indicate that dietary changes have only a modest influence on Lp(a), and often in a direction opposite to that observed for LDL-C concentrations [53]. Fig. 4 summarizes the factors affecting Lp(a) levels in various populations.

### 4.4 Elevated Lipoprotein(a) Levels and Cardiovascular Outcomes: Insights From Recent PCI Studies

Reports examining Lp(a) levels in relation to cardiovascular events have shown the profound repercussions Lp(a) has on clinical outcomes in different cohorts of patients undergoing PCI. Each of these reports has provided insights into the potential clinical implications of Lp(a) levels, covering both in-hospital impact and long-term prognostic implications. There were similarities and divergences in study design and patient populations across reports; however, all efforts have demonstrated Lp(a) as a significant independent risk factor for cardiovascular events.

Sami *et al*. [16] focused on patients with ST-Elevation myocardial infarction (STEMI) undergoing primary PCI. They found that patients with Lp(a) levels ≥30 mg/dL had significantly worse cardiac function. This was evidenced by lower left ventricular ejection fractions (average 45.67% vs. 51.02% in those with lower Lp(a) levels) and higher rates of acute cardiac failure, reinfarction, and in-hospital mortality. Notably, they identified a cut-off value of 24.55 mg/dL for Lp(a), which demonstrated high sensitivity (90.9%) and specificity (91.7%) in predicting adverse outcomes.

In contrast, Liu *et al*. [20] examined a cohort of 4078 patients with stable CAD over a nearly five-year follow-up period. Their findings revealed a substantial 2.1-fold increase in cardiovascular event rates among patients with elevated Lp(a) levels (≥30 mg/dL). Specifically, higher Lp(a) levels were associated with an increased hazard of cardiovascular death (hazard ratio [HR] = 1.9) and stroke (HR = 2.0). This study also demonstrated that adding Lp(a) levels to established risk prediction models significantly improved their accuracies.

Wu *et al*. [21] and Hu *et al*. [18] both investigated patients with ACS. Wu *et al*. [21] discovered that patients with ACS with Lp(a) levels ≥30 mg/dL had increased odds of severe coronary lesions and adverse in-hospital outcomes. Hu *et al*. [18], on the other hand, focused on the combined effect of Lp(a) and GRACE scores. They observed that patients with both high Global Registry of Acute Coronary Events (GRACE) scores and high Lp(a) levels experienced nearly double the risk of cardiovascular events (HR = 1.94) compared to those with lower Lp(a) levels.

Saeki *et al*. [14] uniquely explored the dynamic changes in Lp(a) levels in 377 ACS patients post-PCI. They identified a biphasic pattern in which Lp(a) levels initially decreased, followed by a subsequent increase. Importantly, they found that a greater early reduction in Lp(a) levels was associated with a higher incidence of major adverse cardiovascular events (MACE). Zhu *et al*. [17] investigated the impact of statin treatment on Lp(a) levels in 488 patients with CAD. Their study revealed that an increase in Lp(a) levels of more than 10.1 mg/dL after statin therapy was associated with a significantly increased risk of MACE (HR = 2.29).

Further support for the role of Lp(a) as an independent risk factor came from studies by Amin *et al*. [15], Yoon *et al*. [19], and Liu *et al*. [22]. Amin *et al*. [15] reported a striking HR of 4.29 for high Lp(a) levels in predicting adverse outcomes, even in patients with well-controlled LDL cholesterol levels. Yoon *et al*. [19] corroborated these findings in a similar patient population with an 18-month follow-up. Liu *et al*. [22] also indicated that the high Lp(a) levels independently predict adverse cardiac events.

In conclusion, the combined findings from the evaluated studies strongly support the role of elevated Lp(a) as an independent predictor of cardiovascular risk across a spectrum of clinical settings. This collective evidence advocates for more routine Lp(a) measurement in clinical practice and highlights the need for further research to develop standardized measurement protocols and targeted interventions to mitigate the risk associated with high Lp(a) levels.

### 4.5 Lipoprotein(a) and Residual Inflammatory Risk (RIR)

Lp(a) is gaining recognition not only as a standalone risk factor for atherosclerotic cardiovascular disease but also as a major player in the RIR [54]. Recent evidence highlights the interconnected roles of Lp(a) and chronic inflammation in atherosclerosis progression and residual cardiovascular risk, even among patients undergoing optimal lipid-lowering therapy. In their comprehensive review, Di Fusco *et al*. [55] elucidated how elevated Lp(a) levels (>125 nmol/L or 50 mg/dL) contribute to pro-inflammatory, pro-atherosclerotic, and pro-thrombotic effects through mechanisms such as oxidized phospholipid-mediated monocyte activation, upregulation of endothelial adhesion molecules (e.g., intercellular adhesion molecule-1 [ICAM-1]), and inhibition of fibrinolysis via competition with plasminogen. The authors also explored shared biological pathways with systemic inflammation, as indicated by biomarkers such as high-sensitivity C-reactive protein (>2 mg/L), and advocated for integrated diagnostic and therapeutic strategies, including PCSK9 inhibitors, gene-silencing agents, and anti-inflammatory agents such as colchicine to mitigate these risks. This perspective supports the pursuit of personalized approaches to further reduce the burden of atherosclerotic cardiovascular disease in populations with high Lp(a) levels.

Lp(a) exhibits a range of detrimental properties, including proinflammatory, proatherosclerotic, and prothrombotic properties. The mechanism by which Lp(a) promotes atherosclerosis involves its penetration into the arterial walls (Fig. 3) [56]. Once there, OxPls induce apoptosis, contributing to the development of plaques prone to rupture. Lp(a) directly promotes inflammation within the arterial wall by inducing monocyte transmigration through blood vessel linings and activating endothelial cells. Inflammation is triggered by the increased number of adhesion molecules (e.g., ICAM-1), and production of the enzyme 6-phophofructo-2-kinase/fructose-2,6-biphosphatase (PFKFB3) [39, 57]. Additionally, specific regions within the apo(a) structure, known as kringle IV (KIV) domains, interact with the beta2-integrin protein Mac-1. This interaction activates s nuclear factor-kappa B (NF-κB), a key regulator of inflammation, ultimately boosting the production of molecules that encourage monocyte adhesion and infiltration into the arterial wall [58]. These inflammatory effects are directly proportional to Lp(a) concentration in the blood, with levels exceeding 125 nmol/L (50 mg/dL), signifying a substantially elevated cardiovascular risk [32].

Addressing the inflammatory aspect of Lp(a) risk is a rapidly developing field. Although statins, commonly used to lower cholesterol, have little effect on Lp(a) concentration, PCSK9 inhibitors provide modest reduction [55]. The most striking therapeutic progress, however, has been observed with the previously mentioned RNA-targeted treatments. Beyond its Lp(a)-lowering capabilities, pelacarsen has also shown evidence of decreasing inflammatory activity and the movement of circulating monocytes across the endothelium [59]. While more extensive investigations are needed to confirm the clinical relevance of these anti-inflammatory actions, they point to a possible double advantage of these novel therapies: they lower Lp(a) levels and simultaneously dampen the associated inflammatory cascade.

### 4.6 Future Treatment of Lipoprotein(a) Beyond Statins

Existing therapies for the management of elevated Lp(a) are of particular interest because, although statins are beneficial in reducing LDL-C levels, there is no evidence that statins reduce Lp(a) levels. In addition, options for therapy are limited to niacin, which can lower Lp(a), but has side effects; and cholesteryl ester transfer protein inhibitors with inconsistent evidence. Current developments in lipid reduction include the advent of PCSK9 inhibitors. PCSK9 inhibitors work by blocking the natural degradation of LDL-C receptors, preventing their destruction by binding to PCSK9 [36, 60]. Antibodies targeting PCSK9 can also decrease Lp(a) concentrations by more than 27% [61]. A new PCSK9 inhibitor, inclisiran, which suppresses gene transcription, demonstrated an average Lp(a) reduction of 14%–22% during phase III clinical studies [62, 63]. Another option, mipomersen, also lowers Lp(a); however, similar to niacin, it presents a notable side effect profile [36]. The procedure known as Lp(a) apheresis is the most potent treatment for familial hypercholesterolemia. This method markedly diminishes both LDL-C and Lp(a), achieving reductions of 60–70% per session, and has been shown to improve cardiovascular outcomes [64]. Germany has approved its use for patients with elevated Lp(a) and progressive CVD, with the German Lipoprotein Apheresis Registry (GLAR) offering robust data supporting its effectiveness [65]. The US Food and Drug Administration (FDA) has permitted Lp(a) apheresis for individuals experiencing documented CVD progression and whose Lp(a) levels exceed 60 mg/dL [66].

The therapeutic landscape for Lp(a) is poised for transformation through the use of emerging RNA-targeted therapies. Antisense oligonucleotides (ASOs) and small interfering RNAs (siRNAs) hold great promise. These agents act on RNA molecules to control gene expression, ultimately influencing protein production and significantly reducing Lp(a) levels [36, 67]. ASOs, exemplified by IONIS-APO(a)Rx and pelacarsen, work by suppressing the synthesis of apo(a), achieving substantial Lp(a) reductions of up to 80% in clinical trials [59]. Pelacarsen has demonstrated notable efficacy in phase II trials, exhibiting infrequent and mild adverse effects, typically limited to reactions at the injection site [68]. A phase III trial, known as Lp(a)-HORIZON, is currently in progress to assess pelacarsen’s ability to reduce cardiovascular events [55, 69]. siRNAs, including OLp(a)siran and SLN360, provide a similarly dramatic Lp(a) reduction of up to 90% by targeting Lp(a) mRNA [70]. Phase I and II studies of Olp(a)siran have proven its capacity to induce sustained decreases in Lp(a) concentrations [71, 72, 73]. SLN360 displayed comparable potential in early phase I trials, producing dose-related Lp(a) lowering and exhibiting a favorable tolerability profile [74]. These innovative RNA-based treatments offer the potential to specifically and effectively manage elevated Lp(a) levels, potentially leading to a reduction in cardiovascular risk.

### 4.7 Limitations and Strengths 

Our systematic review has certain limitations that deserve careful analytical attention. First, demographic constitution provides a major limitation; since many of the studies included predominantly Asian populations, generalization to other ethnic groups cannot be safely assumed, given that there are well-documented ethnic disparities in Lp(a) levels and cardiovascular risk profiles.

Another major drawback is the heterogeneity of the methodology. The Lp(a) cut-off values employed were, for the most part, different in the studies included, ranging from 24.55 mg/dL to 50 mg/dL, making direct comparisons rather difficult. Another confounding factor in these studies is the measurement of Lp(a) levels. Some studies measured Lp(a) levels at baseline, others measured post-PCI changes, whereas Saeki *et al*. [14] measured dynamic changes over time, which may represent different pathophysiological processes.

Among the limitations inherent in the study design is the retrospective nature of many studies. This may have led to selection bias and incomplete data collection. The heterogeneity of outcome measures presents considerable issues: some of them defined MACE differently, while follow-up varied from just hospitalization duration to 7.4 years post-inclusion. Hence, it is difficult to establish consistent prognostic thresholds.

The sample sizes showed a wide variation in number, from 70 to 12,064 patients. This could hamper the statistical interpretation of smaller studies and favor larger studies. In addition, most studies did not use an established protocol for measuring Lp(a), thereby bearing possibilities for analytical variability affecting the true validity of the association supposedly verified.

Confounding variables were not controlled in a consistent manner across studies; some studies adjusted for traditional cardiovascular risk factors, while others provided minimal adjustment for significant covariates, such as inflammatory markers, renal function, or genetic factors, which may impact both Lp(a) levels and cardiovascular outcomes.

The strength of our review lies in the synthesis of this varied evidence. By examining a wide range of studies, we highlight a crucial pattern: Lp(a) consistently emerges as a valuable prognostic indicator. This collective evidence provides a compelling argument for the standardization of Lp(a) measurement in clinical practice. It emphasizes the need for clear, universally accepted cut-off points to identify individuals at increased risk. Ultimately, our findings inform future research directions and emphasize the potential for Lp(a)-targeted interventions to improve cardiovascular risk prediction beyond conventional methods.

## 5. Conclusion

This study underscores the critical role of Lp(a) as a potent biomarker for predicting recurrent cardiovascular events in patients with ACS who have undergone PCI. The constant correlation between high Lp(a) levels and negative outcomes underscores the need for regular monitoring and focused Lp(a) management to reduce residual cardiovascular risk, even in the face of limitations in ethnic diversity and methodological variation. This involves prioritizing high-risk populations (e.g., those with Lp(a) >50 mg/dL and high GRACE scores) for new treatments, such as pelacarsen, and standardizing the units to nmol/L.

## Availability of Data and Materials

All data generated or analyzed during this study are included in this published article.

## Supplementary Material

Supplementary material associated with this article can be found, in the online version, at https://doi.org/10.31083/RCM42784.
